# Effects of walking-induced fatigue on gait function and tripping risks in older adults

**DOI:** 10.1186/1743-0003-11-155

**Published:** 2014-11-15

**Authors:** Hanatsu Nagano, Lisa James, William A Sparrow, Rezaul K Begg

**Affiliations:** Institute of Sport, Exercise and Active Living (ISEAL), Victoria University, Ballarat Rd., Footscray, Melbourne, Victoria Australia

**Keywords:** Minimum foot clearance, Fatigue, Swing phase trajectory, Ageing, Treadmill walking, Tripping

## Abstract

**Background:**

Fatigue and ageing contribute to impaired control of walking and are linked to falls. In this project, fatigue was induced by maximum speed walking to examine fatigue effects on lower limb trajectory control and associated tripping risk and overall gait functions of older adults.

**Methods:**

Eleven young (18–35 years) and eleven older adults (>65 years) conducted 5-minute *preferred* speed treadmill walking *prior to* and *following* 6-minute maximum fast walking. Spatio-temporal gait parameters and minimum foot clearance (MFC) were obtained. Maximal muscle strength (hamstrings and quadriceps) was measured on an isokinetic dynamometer. Heart rate (HR) and rating of perceived exertion (RPE) assessed physiological effort and subjective fatigue. Physiological Cost Index computed walking efficiency.

**Results:**

Fatigue due to fast walking increased step length, double support time and variability of step width. Only older adults reduced MFC due to fatigue. A trend of longer double support with greater MFC was found in the non-dominant limb. Lower walking efficiency was characterised as the ageing effect. Older adults did not increase HR during fast walking but higher RPE scores were observed.

**Conclusions:**

Older adults can increase tripping risk by 6 minutes of fast walking possibly by both impaired walking efficiency based on cardiac capacity and higher perceived fatigue due to elevated caution level. Regardless of age, increased step width variability due to fatigue was observed, a sign of impaired balance. Longer double support and greater MFC observed in the older adults’ non-dominant limb could be an asymmetrical gait adaptation for safety.

**Electronic supplementary material:**

The online version of this article (doi:10.1186/1743-0003-11-155) contains supplementary material, which is available to authorized users.

## Background

Walking is critical to quality of life and independence because almost all everyday activities require the capacity to move through the environment to allow useful interactions with people or objects. With ageing walking patterns change due to declines in cognitive and sensorimotor processes associated with reduced muscle activation, impaired balance and loss of co-ordination [1]. One critical factor closely associated with the task of walking itself is ‘fatigue’, which may discourage physical activity and further compromise safe progression [2, 3]. The aim of the present research was to investigate whether walking-induced fatigue increases older adults’ falls risk during locomotion.

Most falls in older adults occur during dynamic activity, such as walking [4] and the risk factors for falling are typically due to mismatched interactions between environmental demands and intrinsic factors associated with ageing [5]. Fatigue is an important intrinsic risk factor for falls because it adversely affects walking and balance [6, 7]. Experimental studies of ageing effects on gait usually incorporate procedures to ensure that fatigue does *not* influence the findings. In contrast, in the everyday environment walking biomechanics are expected to be influenced by fatigue, which may be further accentuated with ageing, such that older adults show a disproportionate decline in gait control and an increased falls risk when fatigued.

**Figure 1:**
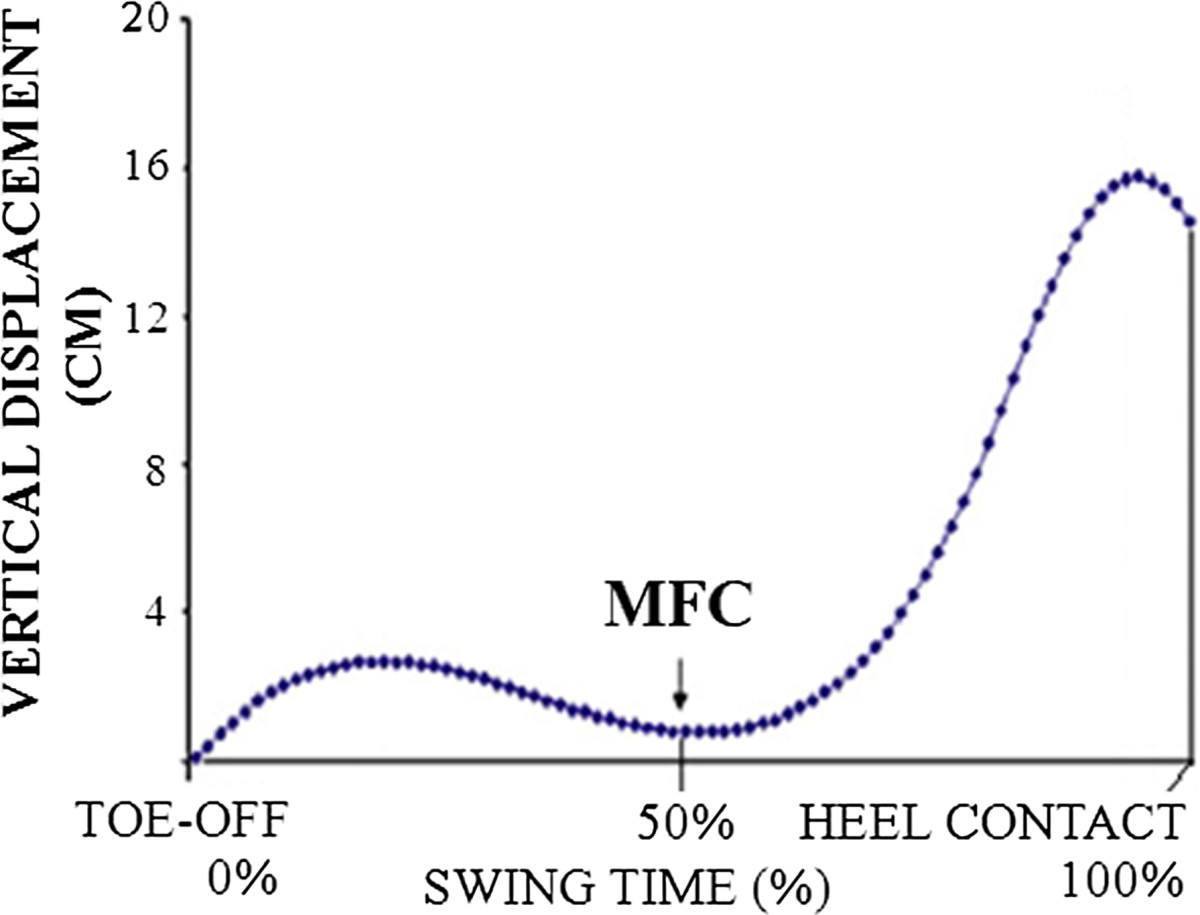
**Vertical displacement of ‘toe’ marker.** During one full swing phase from toe-off to heel contact of the ipsilateral limb. MFC defined as the local minimum foot (toe)-ground clearance between the two maximum swing foot-ground clearances.

Approximately one third of community-dwelling people aged 65 years and above and half of those aged 80 years and over fall at least once a year [8–10]. Falls are the leading cause of injuries and this high falls incidence is associated with elevated morbidity and mortality rates, with the financial cost in Australia an estimated $3 billion annually [11]. Irrespective of the specific interaction of risk factors, it has been found that tripping is responsible for more than one third of falls during locomotion [5, 9, 10, 12]. Tripping occurs during the swing phase of walking when the foot fails to clear either frequently occurring ground irregularities or obstacles. Foot-ground clearance is particularly critical at the swing phase event described as Minimum Foot Clearance (MFC) (see Figure 1). During normal unobstructed gait MFC is minimal, approximately 1-2 cm [13]. MFC occurs approximately halfway through the swing phase, at which time the foot’s horizontal velocity is maximal and, as a consequence, unanticipated contact with the walking surface or an obstruction at this point will be forceful. Tripping risk can be minimised either by increasing MFC or attaining more consistent swing foot control over multiple gait cycles, by reducing MFC variability [13].

In the present project the question was how fatigue induced by fast walking would influence older adults’ overall gait patterns and in particular, foot-ground clearance. Gait adaptations to compensate balance due to fatigue may include providing wider medio-lateral base of support to improve balance and taking longer time in double support to allow more step-to-step correction of minor balance perturbation. As less consistent step width control was previously found as a risk factor for balance loss [14], fatigue effects on balance should be also examined from this perspective.

Research into older adults’ gait has usually either reported unilateral results or the combined data from both limbs but it is increasingly recognised that walking becomes “asymmetrical” with ageing. Gait asymmetry in older people is accentuated when gait control is challenged but there are no previous reports of fatigue effects on gait asymmetry and falls risk [15, 16]. With advanced age different roles appear to be assigned to the dominant and non-dominant lower limbs [15–17]. In the present experiment gait variables for the two limbs were analysed independently to demonstrate any differential effects of fatigue on dominant- and non-dominant foot-ground clearance (MFC) and progression-related variables reflecting balance control.

Experimentally-induced fatigue during static contractions have been useful for identifying localised muscle fatigue effects on gait, such as impaired balance control and joint position sense [6]. The research protocols used to induce this type of fatigue, such as a repeated Sit-to-Stand tasks or isokinetic contractions e.g. [18, 19] are not everyday activities. Given that walking is the most commonly performed activity of daily living it was considered important to investigate specifically the fatigue effects of walking on gait variables. Previously used protocols to induce walking-based fatigue have been limited to *preferred* speed walking for prolonged periods between 20 minutes and 3 hours [20, 21]. The motivation for the present study was to determine whether fatigue induced by more intensive short duration walking activity would negatively affect foot-ground clearance. Consistent with this aim, a previously validated technique [22, 23] was employed whereby participants were fatigued by self-selected *fast* walking on a treadmill for a shorter time (i.e., 6 minutes). It was anticipated that short duration treadmill walking would induce fatigue, given previous reports that treadmill walking presents a greater challenge to older adults than overground walking at the same speed [24].

Fatigue was hypothesised to reduce the capacity for consistent fine-endpoint (foot) control reflected in increased variability of MFC and other spatio-temporal gait parameters. MFC was also expected to reduce with fatigue, further increasing tripping risk. To secure balance compensatory gait adaptations were anticipated, specifically greater step width and prolonged double support following fatigue. Finally, fatigue effects and limb-dominance were investigated guided by the hypothesis that in older adults the non-dominant limb’s supporting role may be accentuated, with increased non-dominant MFC emerging as an neuromotor control strategy to prevent tripping [15, 16].

## Methods

### Participants

The participants were 11 young (30 years ±4.3) and 11 older adults (74.2 years ±4.6) with anthropometric characteristics summarised in Table 1. One participant from both the young and older groups were classified as left limb-dominant, determined using established procedures [17]. All were free of musculoskeletal, orthopaedic or neurological conditions that might impair normal locomotion. Older participants lived independently in the local community and were able to walk unaided. Young participants were recruited from the Victoria University community. All participants provided informed consent using procedures approved and mandated by the Victoria University Human Research Ethics Committee.

**Table 1:** Subject characteristics, walking velocity and fatigue indicators; ageing effects: *p < .05, **p < .01, condition effects: † p < .05; †† p < .01, limb effects: ¶ p < .05

			Young	Older
Anthropometric characteristics	Age (yrs.)		**30.0 ± 4.3	**74.2 ± 4.6
	Height (m)		*1.73 ± 0.07	*1.64 ± 0.08
	Limb Length (m)		0.80 ± 0.05	0.79 ± 0.06
	Body Mass (kg)		69.0 ± 8.1	70.6 ± 10.4
Walking Velocity (km/h)	*Preferred (Baseline/Post)*		†† 4.20	3.59
	*Fast (Fatiguing)*		** †† 7.05	**5.15
Maximal muscle strength (%) Change (Baseline and Post-fatigue)	Hamstring	*Dom*	-1.88	¶ -4.84
		*Non*	-4.48	¶ 5.20
	Quadriceps	*Dom*	-1.06	¶ 7.12
		*Non*	-8.64	¶ -4.55
Heart rate (beats/min)	*Resting*		77.3 ± 9.4	71.3 ± 11.9
	*Baseline*		†† 87.6 ± 13.9	95.5 ± 21.0
	*Fatiguing*		** †† 121.3 ± 14.3	**109.0 ± 20.6
	*Post*		†† 97.4 ± 16.5	95.7 ± 20.6
PCI (beats/m)	*Baseline*		* †† 0.14	*0.43
	*Fatiguing*		* †† 0.37	*0.46
	*Post*		* †† 0.28	*0.44
Borg Scale	*Baseline*		†† 7.9 ± 1.6	†† 9.3 ± 2.0
	*Fatiguing*		†† 12.8 ± 1.3	†† 12.1 ± 2.2
	*Post*		†† 8.1 ± 1.6	†† 8.3 ± 1.6

### Procedures

Toe and heel positions during treadmill walking were measured using an Optotrak (Northern Digital Inc, Canada) 3D motion capture system (100 Hz). An infrared-light emitting diode was placed on 5^th^ metatarsal head to record the vertical swing toe trajectory. Movement of the proximal inferior surface of shoe-outer sole (i.e. heel) was monitored using the virtual marker function [25]. 3D position data were low-pass filtered (6Hz); the filtered kinematic data were then used to define heel contact and toe-off [26]. Vertical toe trajectory from toe-off to heel contact was monitored to identify MFC (Figure 1).

Participants initially walked on the treadmill to familiarise with the task (including the safety harness) and preferred walking speed (PWS) was determined. To establish PWS the participant’s Froude velocity (Vf) was first calculated.

1 $$Vf=\sqrt{Rg}$$

(where R = the length from the greater trochanter to the lateral malleolus and g = gravity).

Participants then walked at 0.42 Vf, the typical comfortable walking speed [27], following which the treadmill speed was increased until the participant perceived the speed to be uncomfortably fast (fast limit). It was then decreased until reported to be uncomfortably slow (slow limit). The mean of three fast and slow limits determined PWS. Each participant completed two 5-minute PWS treadmill walking conditions: Pre-fatigue (Baseline) and Post-fatigue. The fatiguing task in the current project was undertaken between Pre- and Post-fatigue, walking at self-selected maximum speed, which can be *safely* maintained for 6 minutes without being exposed to health risks such as falls and hyperventilation [28].

### Gait parameter definitions

Gait parameters, including spatio-temporal parameters and MFC, were collected only during PWS walking, namely Pre-fatigue (Baseline) and Post-fatigue. Gait parameters were obtained for dominant and non-dominant limbs separately. Spatio-temporal data were summarised using the mean ± standard deviation with dominance of the spatio-temporal parameters following the *lead* limb at heel contact. Step length was anterior-posterior displacement between successive contralateral heel contacts. Normalisation was applied to exclude potential effects of limb length, as follows;

2 $$\mathrm{Normalised}\;\mathrm{Step}\;\mathrm{Length}\;\left(\mathrm{unitless}\right)=\frac{\mathrm{Step}\;\mathrm{Length}\;\left(m\right)}{\mathrm{Limb}\;\mathrm{Length}\;\left(m\right)}$$

(where limb length was measured from the greater trochanter to lateral malleolus).

Double support time was the interval between heel contact and contralateral toe-off, normalised to step time, from heel contact to contralateral heel contact.

3 $$\begin{array}{l} \mathrm{Normalised}\;\mathrm{Double}\;\mathrm{Support}\;\mathrm{Time}\;\left(\%\right)\\ \;=100\left(\%\right)\times \frac{\mathrm{Double}\;\mathrm{Support}\;\mathrm{Time}\;\left(s\right)}{\mathrm{Step}\;\mathrm{Time}\;\left(s\right)} \end{array}$$

Medio-lateral displacement between successive heel contacts defined step width [15].

MFC was the lowest vertical toe displacement during mid-swing between the two peak clearances (Figure 1). Median ± inter-quartile range described MFC data [13].

### Maximal muscle strength estimation

To identify any decrements due to fatigue maximal knee extensor and flexor torque was measured using a Biodex System II isokinetic dynamometer (Biodex Medical Systems, Inc.) both prior to treadmill walking and on completion of the gait tests. Participants performed 5 continuous full-range knee flexion and extension contractions at 60°/s with maximal strength represented by the mean of the 5 peak torques from each trial [6]. Changes (%) in peak knee extension/flexion torques from Baseline to Post-fatigue were recorded.

### Fatigue assessments

Two types of fatigue assessment were taken every minute to accommodate the multidimensionality of fatigue and averaged to represent each walking condition including Baseline, Post-fatigue and also the 6-minute fast-walking. Subjective ratings of perceived exertion (RPE), which have been established as valid indicators of exercise intensity were measured using the Borg scale, ranging from 6 (“no effort at all”) to 20 (“maximal effort”) [29].

A Polar Heart Rate Monitor recorded heart rate (HR) including resting HR to estimate physiological workload, which has been shown to correlate with RPE [30]. HR data combined with walking speed were used to determine a Physiological Cost Index (PCI) in beats per meter walked with greater efficiency associated with lower PCI [31].

4 $$PCI=\frac{\mathrm{Walking}\;HR-\mathrm{Resting}\;HR}{\mathrm{Walking}\;\mathrm{Speed}}\;\left(\mathrm{beats}/m\right)$$

### Design and analysis

Different mixed model repeated measures Analysis of Variance (ANOVA) designs were applied as indicated (SPSS 20.0, SPSS Inc., Chicago, IL, USA). Computed *P*-values of .05 or less were accepted as statistically significant.

(i) PWS and fast walking speed: A 2 X 2 (age X speed) design, age (Young, Older) the between-subject factor, speed (PWS, fast walking speed) the within-subject factor.
(ii) Spatio-temporal parameters and MFC: A 2 X 2 X 2 (age X condition X limb) design, age (Young, Older) the between-subject factor, condition (Baseline, Post-fatigue) and limb (dominant and non-dominant) the within-subject factors.
(iii) Changes (%) in peak knee extension/ flexion torques from Baseline to Post-fatigue: A 2 X 2 (age X limb) design, age (Young, Older) the between-subject factor, limb (dominant and non-dominant) the within-subject factor.
(iv) HR, PCI and RPE: A 2 X 3 (age X condition) design, age (Young, Older) the between-subject factor, condition (Baseline, 6-minute Fatigue, Post-fatigue) the within-subject factor.

## Results

PWS was not differentiated between the age groups, but maximum walking speed during the fatiguing task was significantly greater in the young group (young 7.05 km/h vs. older 5.15 km/h), indicated by an age X speed interaction (F_(1, 20)_ = 16.9, p < .01).

### Spatio-temporal parameters

**Figure 2:**
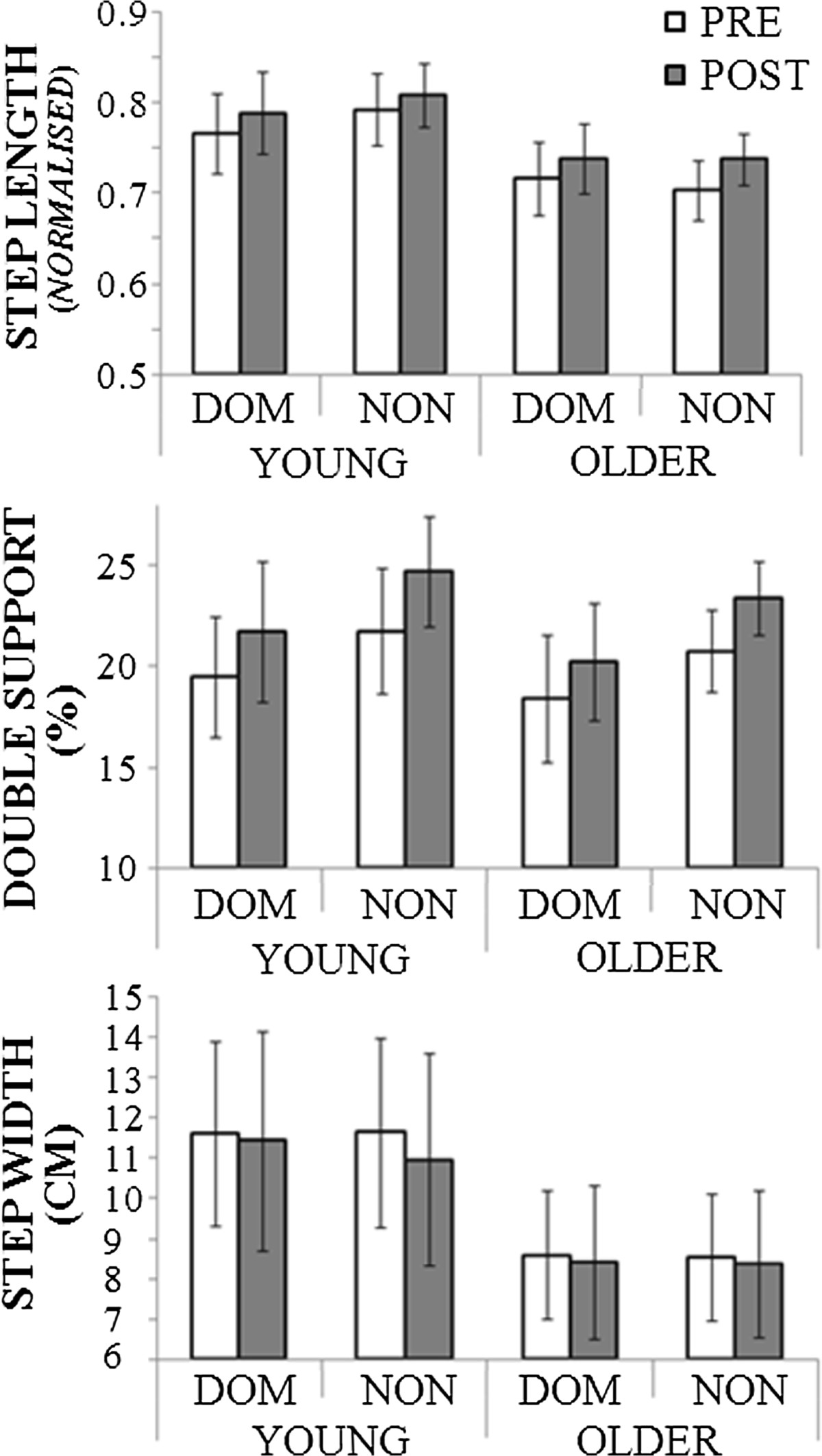
**Spatio-temporal gait parameters comparison in Baseline (pre) and post-fatiguing (post) condition.** Dominant (DOM) and non-dominant (NON) parameters separately.

Ageing, limb and fatigue effects on spatio-temporal parameters are presented in Figure 2. Step length was not significantly influenced by ageing despite a trend for greater step length in the young. Step length increased after the fatiguing task (F_(1, 20)_ =18.4, p < .01): Baseline (0.74) and Post-fatigue (0.77). Proportion of double support was greater in the non-dominant limb by 2% than the dominant side (F_(1,20)_ =4.6, p < .05). In Post-fatigue, 2% increase in double support proportion relative to Baseline was also observed (F_(1,20)_ =4.4, p < .05). Step width variability (SD) increased in Post-fatigue compared to Baseline by 0.3 cm (F_(1, 20)_ =29.1, p < .01) and older adults showed 0.8 cm less variable step width (F_(1, 20)_ =6.3, p < .05).

### MFC

**Figure 3:**
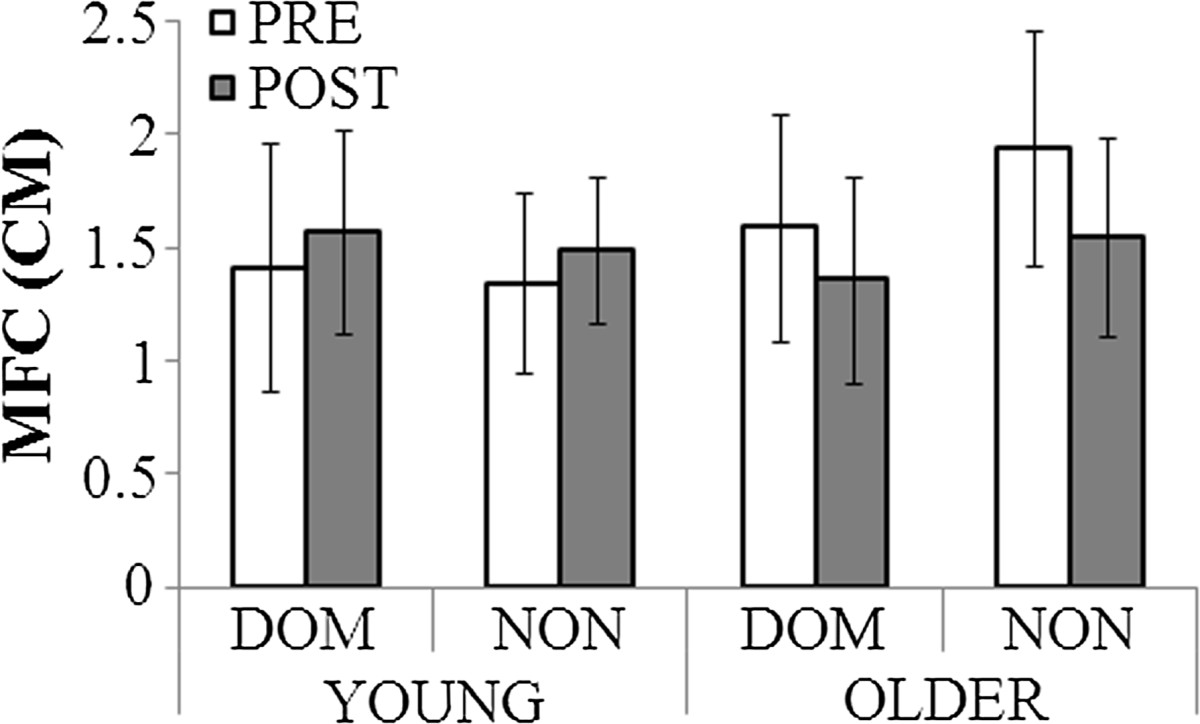
**Minimum Foot Clearance (MFC) data.** Dominant (DOM) and non-dominant (NON) parameters separately.

After the fatiguing task, the older adults significantly reduced MFC by 0.31 cm (F_(1, 20)_ =6.8, p < .05), but their MFC variability also significantly reduced (F_(1,20)_ =6.9, p < .05) (Figure 3). The older group showed a non-significant trend toward asymmetrically greater MFC in the non-dominant limb. Asymmetrically lower MFC variability in the non-dominant limb was the only effect specifically seen in the young group (F_(1,20)_ =5.7, p < .05).

### Maximal muscle strength

Changes in maximal muscle strength due to fatigue are summarised in Table 1. Despite an overall trend of reduced torques in the Post-fatigue, the non-dominant limb of older adults showed an increase (F_(1,20)_ =4.9, p < .05). For older adults’ quadriceps, greater torque production was identified in the dominant limb (F_(1,20)_ =6.6, p < .05).

### Fatigue Assessments: HR, PCI, RPE

Table 1 also presents the results of fatigue assessments. Higher HR was observed in the younger group during the fatiguing task compared to the older participants (F_(2,19)_ =15.1, p < .01). No differences were obtained during PWS conditions. PCI analysis revealed that the older group generally had higher scores (F_(2,19)_ = 5.9, p < .05). Older participants did not change their PCI scores as much as the young across the three conditions. The young group’s PCI scores were at least doubled in the fatiguing and Post-fatigue conditions compared to their Baseline PCI (F_(2,19)_ =11.3, p < .01). Both groups increased RPE during the fatiguing task (F_(2,19)_ =43.4, p < .01), but no significant ageing effects were found.

## Discussion

Similar fatigue effects on gait parameters were seen in both age groups. Following 6 minutes of fast treadmill walking both groups increased step length to maintain the target walking speed, consistent with previous findings [21]. As hypothesized, the proportion of double support within step time increased following the fatiguing task, a response that would promote stability but it also decreased step frequency, requiring greater step length to maintain walking speed. Contrary to expectation, step width did not increase in response to fatigue, possibly because such adaptations may be functionally suboptimal to maintain PWS on a treadmill. Although no wider base of support was confirmed, both age groups increased step width variability in Post-fatigue, as also reported in earlier work [18], and this effect is widely accepted as an indicator of reduced stability and greater falls risk [14].

From bi-lateral analysis of spatio-temporal parameters, 2% greater non-dominant double support (from non-dominant heel contact to dominant toe-off) was discovered. Before the dominant limb commences swing phase, body stabilisation can be considered important for the non-dominant stance limb. Prolonged double support is effective for micro-balance adjustment prior to the more destabilising swing phase of the dominant limb, consistent with ‘functional asymmetry’ interpretations of the non-dominant limb’s role in securing gait stability [15–17].

Further evidence of functional asymmetry was identified in the trend toward asymmetrically greater MFC among the older adults, as also previously reported [16]. This could be an attempt by the motor system to implement a safe foot trajectory on the non-dominant side. MFC is, as already highlighted, the critical gait variable for predicting tripping risks due to the swing foot-contact with surface obstacles. Regardless of age, limb or walking conditions, MFC data in the current study was in the typical range (1.0 cm- 2.0 cm); a result similar to data in a systematic review by Barrett et al. [32]. The most striking finding is, however, in showing that with ageing swing foot-ground clearance at MFC in the everyday environment is expected to reduce with fatigue due to as little as 6 minutes of fast walking, increasing the risk of tripping over obstacles or frequent small irregularities in the walking environment.

Age-specific fatigue effects were thus found in lower swing foot-ground clearance at MFC and accordingly, the greater tripping risk can be predicted if fatigue was induced by fast walking. It is, however, interesting to review the protocol used to induce fatigue (i.e. fast walking) and results of the fatigue assessments in detail particularly for older individuals based on both HR and RPE. As highlighted in Table 1, older adults’ self-selected maximum treadmill walking speed was not significantly greater than their PWS. Similarly, no remarkable increment in HR in response to the fatiguing task was identified for older adults.

In terms of walking efficiency, however, older adults demonstrated more heart beats per one meter progression than their young counterparts as shown in higher PCI scores (Table 1). This may suggest that older adults should be more prone to fatigue than the young when walking. In addition to physiological fatigue, perceived fatigue during ‘fast walking’ was found for the older group as shown in increased RPE. It is, therefore, also possible that they may have developed caution-based fatigue induced by fast treadmill walking [24]. Age-specific increased tripping risk due to fatigue can be accordingly caused by impaired walking efficiency and elevated alertness in walking. The fatigue-inducing protocol in the current research was modelled on an established method for clinical gait assessment, the ‘six minute walk test’, that has been used to monitor gait performance in healthy and pathological populations based on the distance walked in six minutes, usually overground [22, 23]. Treadmill walking was, however, suitable for collecting the more extensive data to reflect longer-term sensory inputs for gait control in both steady-state walking and the fatigue condition free of gait adaptations secondary to changes in walking speed.

Fatigue effects on older adults’ gait is the major finding of the study and the link between reduced MFC and the unique pattern of fatigue onset in older participants has been discussed above. Gait patterns have been reported to change due to local muscle fatigue experimentally induced using isokinetic knee flexion/extension [19] but the question remained as to whether similar fatigue effects would appear following fast walking for 6 minutes. Isokinetic contraction capacity reduced for young individuals due to fatigue but surprisingly for the older people maximum torque production in the non-dominant hamstring and dominant quadriceps increased. Warm-up effects from treadmill testing, familiarity with the isokinetic contraction protocol or an increased proportion of endurance (Type I) muscle fibers with ageing could possibly account for increased strength post-testing. These explanations cannot, however, account for asymmetry in the older adults’ torque production between the two limbs. To further understand the neuromotor processes by which fatigue influences gait control with ageing, muscle activation in the dominant and non-dominant limbs could be compared using electromyography.

In this study fatigue was induced using relatively short duration (6 min) fast walking but this protocol should be reconsidered as a fatiguing intervention for older adults. It was expected that treadmill walking would be fatiguing for older adults but the present findings suggest that self-selected fast walking on a treadmill does not allow maximal physical effort due to caution. A more extended preferred speed trial could be investigated and other fatigue-inducing conditions resembling everyday activity, such as grade walking, may also be tested. In future work it would also be informative to determine whether the mechanical energy cost of walking increases with fatigue. Fatigue may, therefore, be considered as both a “cause and effect” phenomenon, in that changes to lower limb control with fatigue may increase the mechanical energy cost of walking at the same speed leading to less economical gait which further fatigues the individual.

## Conclusions

Fatigue induced by 6 minutes of fast walking increased older adults’ tripping risk due to reduced MFC. Declines in walking efficiency based on cardiac capacity and higher perceived exertion were identified in the older individuals, suggesting age-associated proneness to fatigue. Greater step width variability was characterised as a fatigue effect common to both young and older adults, a gait characteristic that reflects balance impairment. It is hypothesised that as postural instability increased with fatigue, the biomechanical strategy to avoid tripping due to reduced foot-ground clearance was to minimise MFC variability. This response appears to be the only biomechanical solution to the motor control problem of maintaining safe ground clearance with increasing fatigue. Biomechanically, dynamic stability is preserved by maintaining the whole body centre of mass within the support base associated with step width and length. Step width variability reflects motor control strategies to maintain medio-lateral stability such that, in an unstable environment, greater response variability in the more distal segments would be required to maintain whole body stability. Age-associated asymmetry in gait was observed such that the older adults’ non-dominant limb had longer double support with increased MFC suggesting that the non-dominant limb’s role was to secure gait by maintaining balance.

## Authors’ original submitted files for images

Below are the links to the authors’ original submitted files for images. Authors’ original file for figure 1 Authors’ original file for figure 2 Authors’ original file for figure 3

## Abbreviations

MFC: Minimum foot clearance
HR: Heart rate
RPE: Rating of perceived exertion
PWS: Preferred walking speed
Vf: Froude velocity
PCI: Physiological cost index
ANOVA: Analysis of variance.
