# Supplementary figures and images for: Effects of walking-induced fatigue on gait function and tripping risks in older adults
Source: J Neuroeng Rehabil. 2014 Nov 15;11:155. doi: 10.1186/1743-0003-11-155 (PMC4253993; doi:10.1186/1743-0003-11-155)

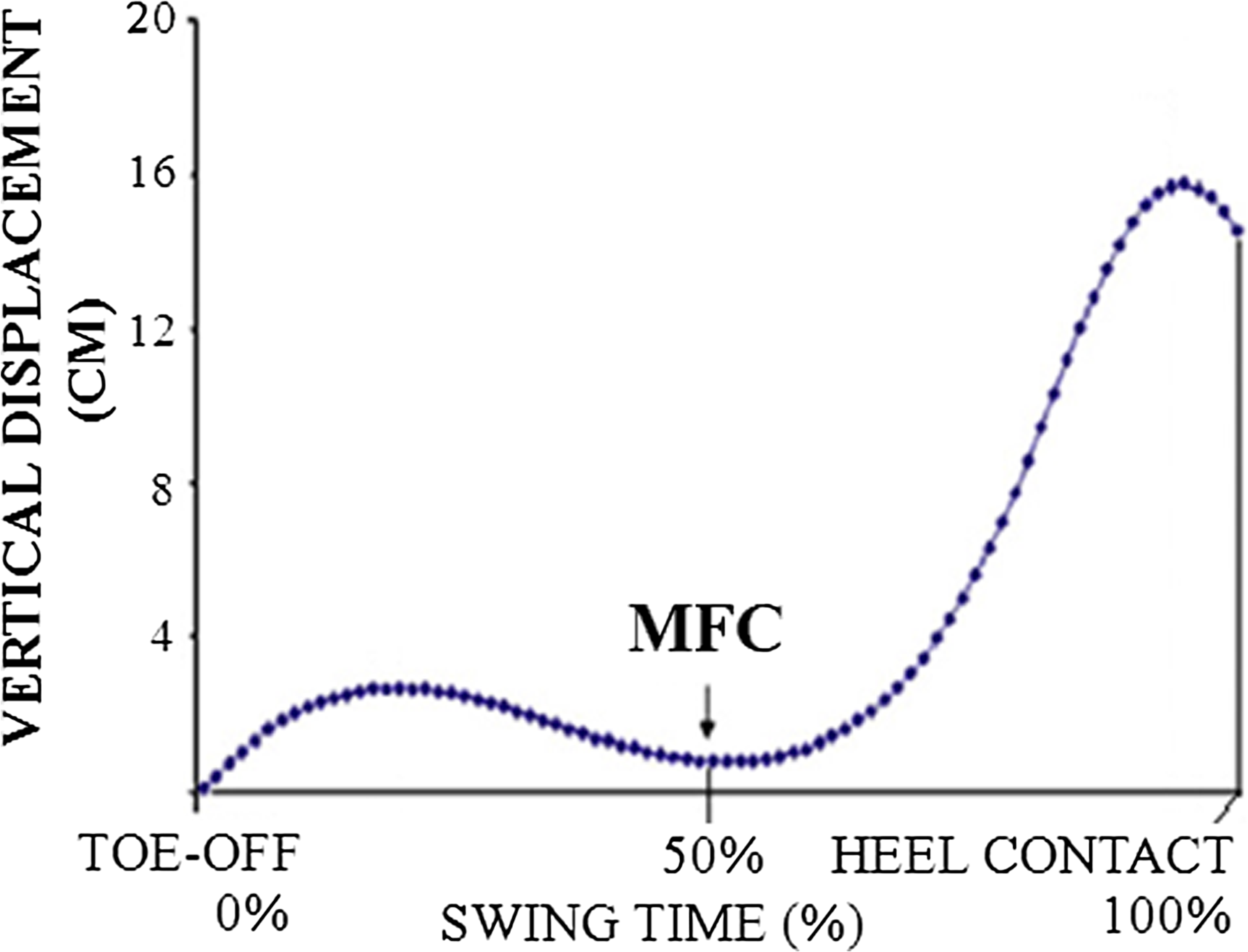

Supplement: Supplementary file 1 — Authors’ original file for figure 1 [file 12984_2014_676_MOESM1_ESM.tif]

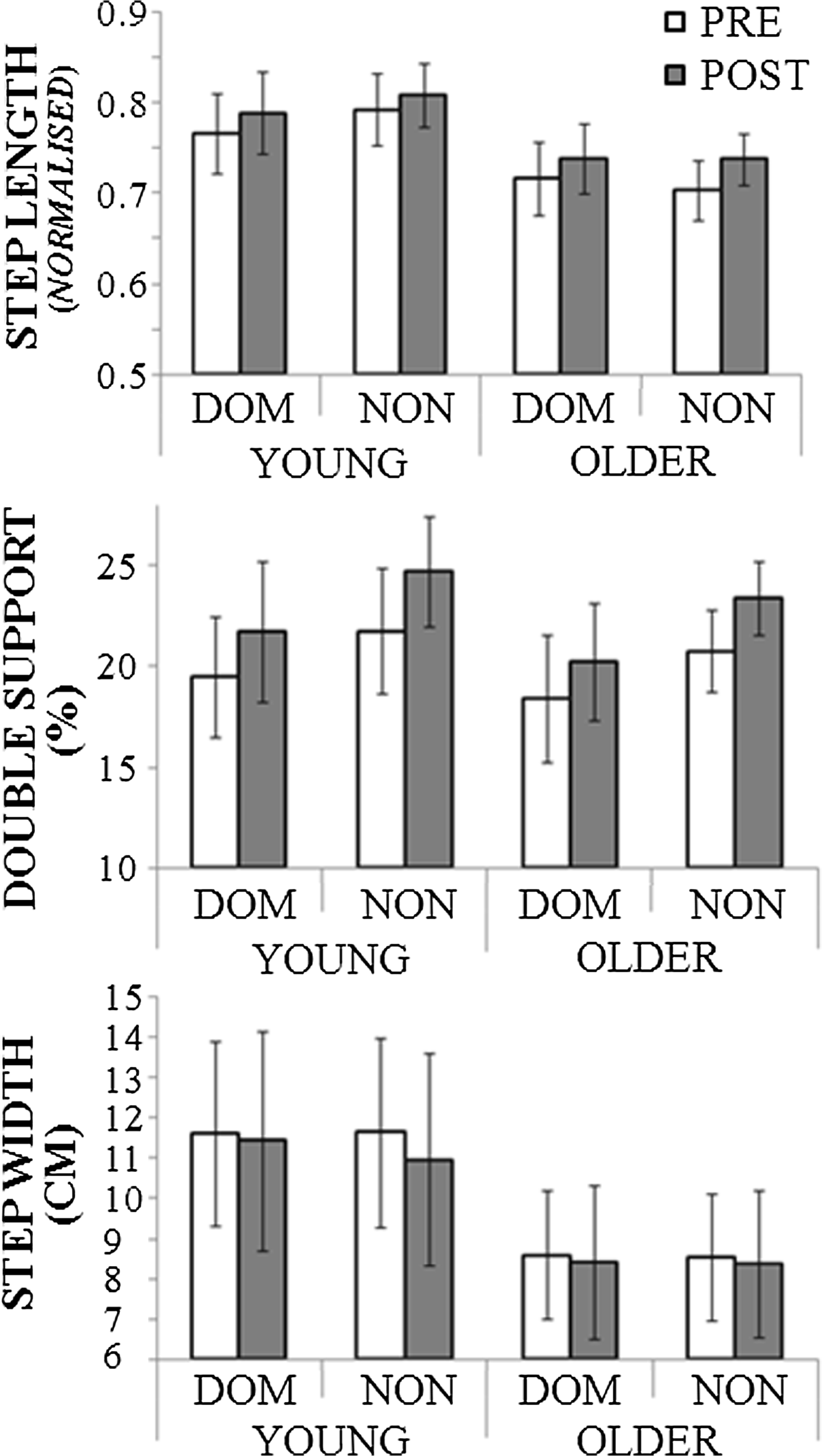

Supplement: Supplementary file 2 — Authors’ original file for figure 2 [file 12984_2014_676_MOESM2_ESM.tif]

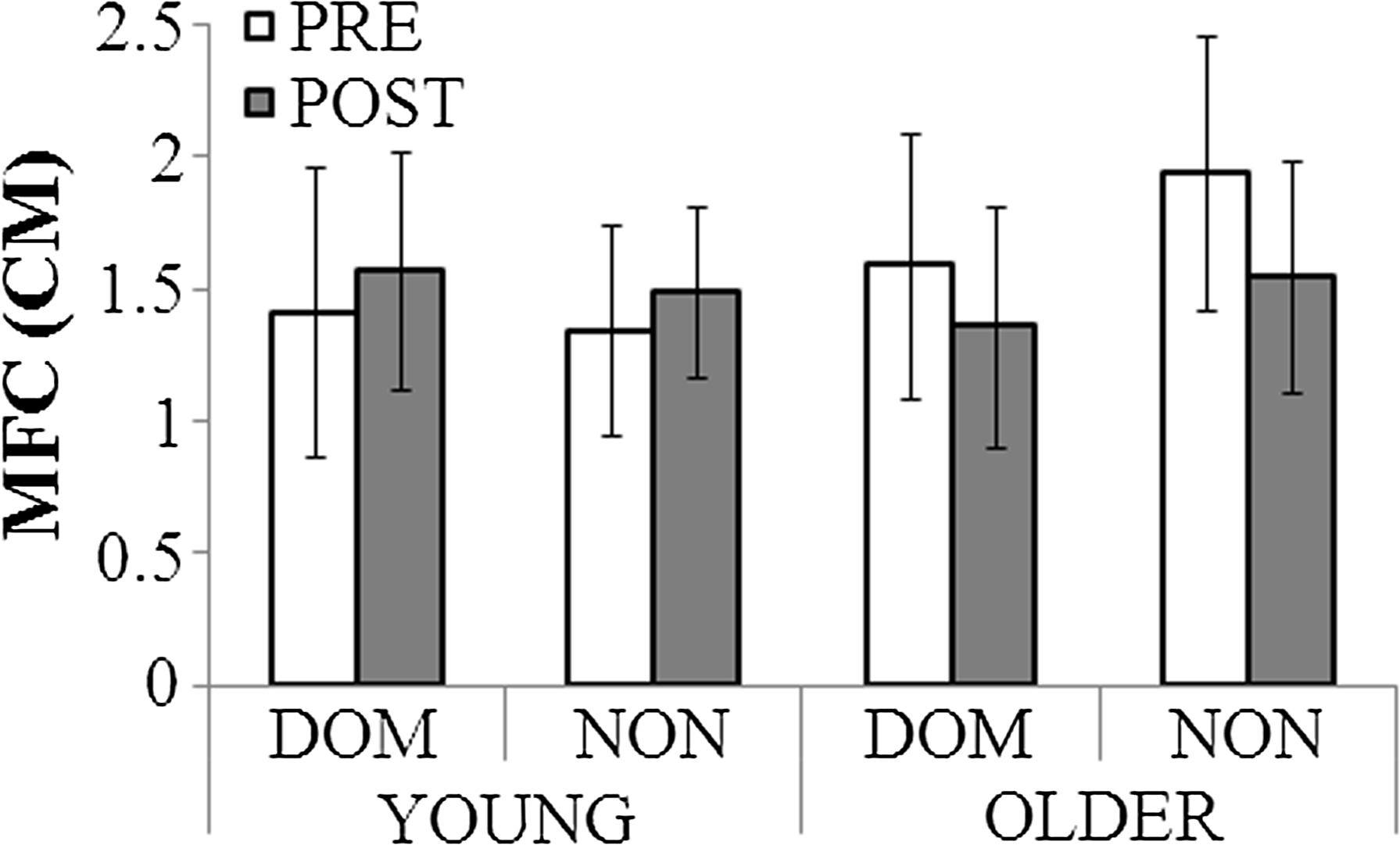

Supplement: Supplementary file 3 — Authors’ original file for figure 3 [file 12984_2014_676_MOESM3_ESM.tif]
